# Supplementary material for: Combining energy efficiency and quantum advantage in cyclic machines
Source: Nat Commun. 2025 Jun 2;16:5127. doi: 10.1038/s41467-025-60179-5 (PMC12130271; doi:10.1038/s41467-025-60179-5)
Supplement: Supplementary file 1 — Supplementary Information [file 41467_2025_60179_MOESM1_ESM.pdf]

Supplementary Information: Combining energy efficiency and quantum advantage in cyclic machines

Waner Hou,[1,2,*] Wanchao Yao,[1,2,*] Xingyu Zhao,[1,2,3] Kamran Rehan,[1,4,†] Yi Li,[1,2] Yue Li,[1,2] Eric Lutz,[5,‡] Yiheng Lin,[1,2,3,§] and Jiangfeng Du[1,2,3,6,¶]

[1]*CAS Key Laboratory of Microscale Magnetic Resonance and School of Physical Sciences, University of Science and Technology of China, Hefei 230026, China*
[2]*Anhui Province Key Laboratory of Scientific Instrument Development and Application, University of Science and Technology of China, Hefei 230026, China*
[3]*Hefei National Laboratory, University of Science and Technology of China, Hefei 230088, China*
[4]*Department of Physics, The University of Haripur, KP Pakistan*
[5]*Institute for Theoretical Physics I, University of Stuttgart, D-70550 Stuttgart, Germany*
[6]*Institute of Quantum Sensing and School of Physics, Zhejiang University, Hangzhou 310027, China*

The Supplementary Information provides details about (I) the implementaion of the theoretical Hamiltonian of the system, (II) the experimental setup, as well as a discussion of (III) the effects of the counterdiabatic driving and (IV) the connection between work output fluctuations and intercycle quantum coherence.

I. THEORETICAL DETAILS

A. Derivation of the Hamiltonian

We experimentally implement the Hamiltonian of a qubit quantum engine system (in units of $\hbar$):

$$H(t) = H_E(t) + H_B + H_{EB}(t), \tag{S1}$$

with

$$H_E(t) = \frac{\Omega}{2}\sigma_x + \frac{v(t)}{2}\sigma_z, \tag{S2}$$

$$H_B = \omega a^\dagger a, \tag{S3}$$

$$H_{EB}(t) = -\frac{\eta\Omega}{2}\sin(\omega t)\sigma_y(a + a^\dagger), \tag{S4}$$

which describes an ensemble consisting of a two-level system and its momentum phonon system. This Hamiltonian can be constructed by the eigen-Hamiltonian of an ion trapped in harmonic confinement with the ion-laser interaction Hamiltonian under rotating-wave approximation (RWA)[1]. The energy of a two-level ion trapped in a harmonic potential is described by the Hamiltonian

$$H_0 = \frac{\omega_0}{2}\sigma_z + \omega_z(a^\dagger a + \frac{1}{2}), \tag{S5}$$

where ω_z is the ion's motional frequency along the z-direction of the potential well, and ω_0 is the unperturbed two-level system (TLS) transition frequency. A laser light field with frequency ω_L and phase ϕ_L induces a perturbation described by the Hamiltonian

$$H_L = \frac{\Omega}{2}\sigma_x(e^{i\eta(a+a^\dagger)}e^{-i(\omega_L t+\phi_L)} + h.c.), \tag{S6}$$

where the Rabi frequency Ω represents the coupling strength between the laser field and the bare two-level atomic transition. An additional term $\exp(ik\hat{x}) = \exp\{i\eta(a + a^\dagger)\}$, where the Lamb-Dicke parameter $\eta = kx_0\cos\theta$, describes the interaction between laser and a trapped ion with additional oscillating motion. With the overall Hamiltonian $H = H_0 + H_L$, a transformation with $U^{spin} = \exp\left(-iH_0^{spin}t\right)$, where $H_0^{spin} = \frac{\omega_0 - v}{2}\sigma_z$, into the interaction picture yields:

$$H_{int}^{spin} = \frac{v(t)}{2}\sigma_z + \omega_z(a^\dagger a + \frac{1}{2}) + \frac{\Omega}{2}(e^{i\eta(a+a^\dagger)}e^{-i(\delta t+\phi_L)}\sigma_+ + h.c.), \tag{S7}$$

where $\delta = \omega_L - (\omega_0 - v)$ denotes the detuning of the laser frequency from the atomic transition. Here, a RWA has been performed and all terms rotating at the sum frequency $\omega_L + (\omega_0 - v)$ are neglected, as they average out over the time scale of δ. Then another transformation with $U^{motion} = \exp\left(-iH_0^{motion}t\right)$, where $H_0^{motion} = (\omega_z - \omega)a^\dagger a = \omega_z' a^\dagger a$, is performed to further simplify the interaction Hamiltonian to:

$$H_{int} = \frac{v(t)}{2}\sigma_z + \omega a^\dagger a + \frac{\Omega}{2}(e^{-i(\delta t+\phi_L)}\sigma_+ \exp\{i\eta(ae^{-i\omega_z' t} + a^\dagger e^{i\omega_z' t})\} + h.c.). \tag{S8}$$

In the Lamb-Dicke regime $\eta^2(2\bar{n} + 1) \ll 1$, where $\bar{n}$ is the average phonon number of the harmonic oscillator, the ion's motional wave packet is confined to an extent which is much smaller than the laser's wavelength. This Lamb-Dicke approximation can further simplify Eq. (S8) by Taylor expanding it to:

$$H_{int} = \frac{v(t)}{2}\sigma_z + \omega a^\dagger a + \frac{\Omega}{2}(e^{-i(\delta t+\phi_L)}\sigma_+\{1 + i\eta(ae^{-i\omega_z' t} + a^\dagger e^{i\omega_z' t})\} + h.c.). \tag{S9}$$

In the experiment, we apply a three-color light field in the same direction to an ion to induce the total Hamiltonian $H_L^{total} = H_L^{carrier} + H_L^{red} + H_L^{blue}$, as shown

in Fig. S1. The frequency and phase relationship between them is $\delta^{carrier} = 0$, $\delta^{blue} = -\delta^{red} = \omega_z^{'}$ and $\phi_L^{carrier} = \phi_L^{blue} = \phi_L^{red} = 0$. An intensity modulation with frequency ω is performed onto the red-blue sideband bicolor light $\Omega^{blue} = \Omega^{red} = \Omega\sin(\omega t)$, while the amplitude is the same with carrier light $\Omega^{carrier} = \Omega$. The main reason for using this intensity modulation function is to ensure that the average phonon number of the harmonic oscillator can also be increased accordingly under the new rotating frame, while the coherence of the spin quantum heat engine itself is unaffected[2,3], so that the work produced by the engine can be distinguished from the noise of background heating. After taking into account all the three frequency components, a RWA is performed, and all terms rotating at $\omega_z^{'}$ and its higher orders are neglected. The total interaction Hamiltonian can be finally modified to:

$$\begin{aligned} H_{int}^{total} &= \frac{v(t)}{2}\sigma_z + \omega a^\dagger a + \frac{\Omega^{carrier}}{2}\sigma_x \\ &+ \frac{\Omega^{blue}}{2}(i\eta\sigma_+ a^\dagger + h.c.) + \frac{\Omega^{red}}{2}(i\eta\sigma_+ a + h.c.) \\ &= \frac{v(t)}{2}\sigma_z + \omega a^\dagger a + \frac{\Omega}{2}\sigma_x - \frac{\eta\Omega}{2}\sin(\omega t)\sigma_y(a + a^\dagger). \end{aligned} \tag{S10}$$

It can be seen that Eq. (S10) is consistent with Eq. (S1). Note that a different ion trap quantum engine, where the motional degree of freedom plays the role of the cold bath, has been discussed in Ref.[4].

B. Shortcut to adiabaticity for the two-level system

For an arbitrary time-dependent isolated Hamiltonian $H_0(t)$ with instantaneous eigenstates $\{|j(t)\rangle\}$ and energies $\{E_j(t)\}$, from Berry's formulation[5], the resulting counterdiabatic Hamiltonian $H_{CD}(t)$ is:

$$H_{CD}(t) = i\sum_j (|\partial_t j\rangle\langle j| - \langle j|\partial_t j\rangle |j\rangle\langle j|). \tag{S11}$$

Note that the eigen-Hamiltonian of the qubit quantum engine is $H_E(t) = \frac{\Omega}{2}\sigma_x + \frac{v(t)}{2}\sigma_z$, and it corresponds to the Landau-Zener model which is well studied in many physical settings[6]. The counterdiabatic Hamiltonian of the Landau-Zener model can be directly written as[5,7]:

$$\begin{aligned} H_{CD}(t) &= \frac{1}{2}\frac{\dot{\Omega}v(t) - \Omega\dot{v}(t)}{\Omega^2 + v(t)^2}\sigma_y \\ &= -\frac{1}{2}\frac{\Omega\dot{v}(t)}{\Omega^2 + v(t)^2}\sigma_y. \end{aligned} \tag{S12}$$

To ensure that the effective Hamiltonian $H_{STA}(t) = H(t) + H_{CD}(t)$ equals the original Hamiltonian $H(t)$ [Eq. (S1)] at the start and end of the protocol, we impose a condition on the form of $v(t)$: $\dot{v}(0) = \dot{v}(\tau/2) = 0$. Here, for simplicity, we select the following time-dependence profile of $v(t) = v_0(\frac{t}{\tau/2})^2(3 - 2\frac{t}{\tau/2})$ to satisfy this boundary condition, while the energy gap of the TLS at the start and end of the protocol is still $E(0) = \Omega$ and $E(\tau/2) = \sqrt{\Omega^2 + v_0^2}$. In the experiment, H_{CD} [Eq. (S12)] can be constructed by adding a laser field with $\delta^{CD} = \delta^{carrier} = 0$, $\phi_L^{CD} = -\pi/2$ and $\Omega^{CD} = \frac{\Omega\dot{v}(t)}{\Omega^2 + v(t)^2}$ [Eq. (S9)].

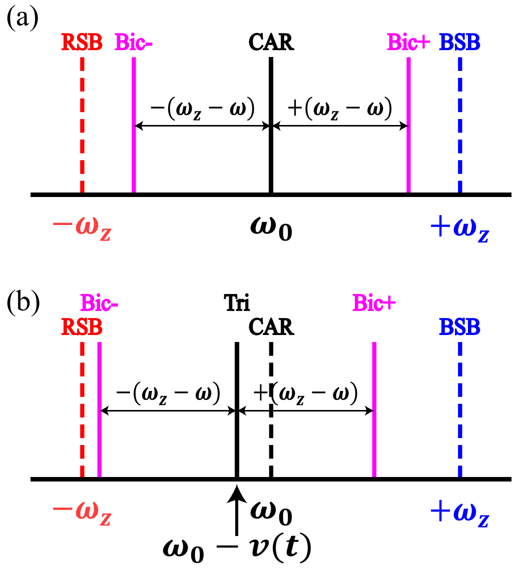

FIG. S1. Illustration of the three-color light field used in the experiment. The dashed lines show the unperturbed frequency of the TLS transition and harmonic oscillator, while the solid lines show the frequency of the three-color light field. (a) The three-color light field at the start ($t = 0$) and end ($t = \tau$) of one Otto cycle. The bichromatic light field, denoted as Bic+ and Bic-, has a symmetric detuning ω from the harmonic oscillator mode ω_z, leading to the transformation in Eq. (S8). (b) The three-color light field at time t during expansion and compression strokes. An additional detuning $-v(t)$ is induced onto the center frequency of the three-color light field, known as a center-line detuning, leading to the transformation in Eq. (S7).

II. EXPERIMENTAL DETAILS

A. Experimental setup and data processing

To simulate such a quantum engine system, we trap a single $^{40}Ca^+$ ion in a linear Paul trap with the ambient magnetic field of 0.538 mT. The harmonic oscillator eigenstates of $^{40}Ca^+$ ion along the z-axis of the potential well, denoted as $\{|n\rangle\}$, are utilized as the eigenstates of the phonon system. Its oscillation frequency within the potential well is denoted as ω_z. For $^{40}Ca^+$ ion, the popu-

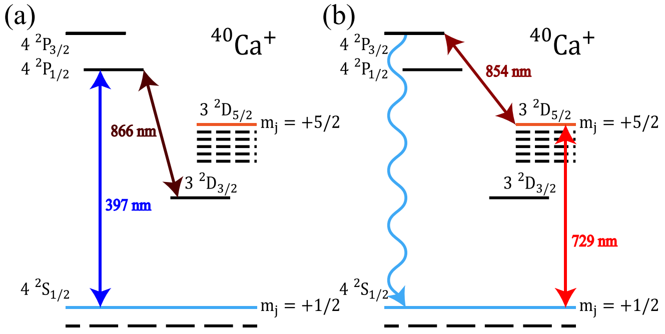

FIG. S2. Illustration of the $^{40}Ca^+$ ion energy levels and transitions. (a) The population of the ground state $S_{1/2}$ is detected via electron shelving. (b) The 854 nm laser is used to quench the metastable state $D_{5/2,m_j=+5/2}$, thus reset the spin state to ground state $S_{1/2,m_j=+1/2}$. The 729 nm laser is used for initial state preparation and engine driving.

lation of ground state $4s\ ^2S_{1/2,m_j=+1/2}$, which is selected as the spin-down state $|\downarrow\rangle$, can be detected by means of electron shelving[8]. Here, 397 nm laser light excites the transition between $S_{1/2} - P_{1/2}$. If the valence electron is in the $S_{1/2}$ state, photons are scattered and collected by a photomultiplier tube (PMT), whereas if the electron is in the $D_{5/2}$ state, which is selected as the spin-up state $|\uparrow\rangle$, no photons are scattered. A laser at 866 nm serves to repump the ion via $P_{1/2} - D_{3/2}$ during electron shelving, thus closing the fluorescence excitation cycle, as shown in Fig. S2(a). For each data point of spin state, the preparation, evolution, and detection are repeated 200 times. We simulate the error of qubit state detection by assuming the normal distribution of the raw data, and sample 200 times from the raw data, leading to an estimation of the standard deviation of the qubit state population.

During the heating and cooling strokes of one Otto cycle, the state of the qubit needs to be reset and prepared in a negligible time to thermalize with the heat baths. In the experiment, the 854 nm laser is used to excite the transition between $D_{5/2} - P_{3/2}$, then the spontaneous emission of $P_{3/2}$ state causes the valence electron to return to $S_{1/2}$ state, thus resetting the spin state to $|\downarrow\rangle$ state, while leaving the phonon state unaffected. Since we select the metastable state $3d\ ^2D_{5/2,m_j=+5/2}$ and ground state $4s\ ^2S_{1/2,m_j=+1/2}$ as the spin-up ($|\uparrow\rangle$) and spin-down ($|\downarrow\rangle$) states respectively, the selection rules ensure that the valence electron in the $D_{5/2,m_j=+5/2}$ state will only be excited to $P_{3/2,m_j=+3/2}$ state, and then deexcite to $S_{1/2,m_j=+1/2}$ state, thus ensuring that no spin population leakage to other sub-levels during the multiple cycles, as shown in Fig. S2(b). After resetting the spin state with a 854 nm laser, when it is needed to prepare the initial spin state according to the temperature of the heat baths, the 729 nm laser pulses of different duration times can be used to adjust the population of

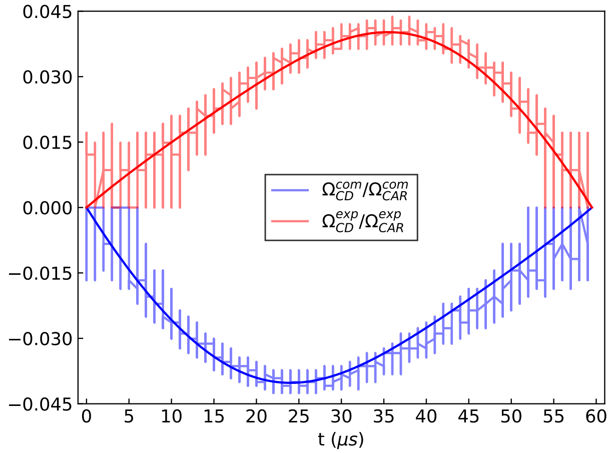

FIG. S3. Measured amplitude proportion of CD laser to carrier laser during the compression and expansion strokes, with $\Omega = 2\pi \times 0.159$ MHz, $\omega_z = 2\pi \times 2.0338$ MHz, $v_0 = \omega = 2\pi \times 0.075$ MHz and $\tau = 119\ \mu$s. Ω_{CD}^{com} is the measured amplitude of CD laser at different times t during the compression stroke, while Ω_{CAR}^{com} is the measured amplitude of carrier laser at the same time t. The measured results during expansion stroke are denoted as $\Omega_{CD}^{exp}/\Omega_{CAR}^{exp}$ correspondingly. The translucent lines with noise are the results of sampling measurements from an oscilloscope. Solid lines represent the corresponding numerical simulations.

$|\uparrow\rangle$ state. In the experiment, for simplicity, we always set the hot equilibrium state to the $|\uparrow\rangle$ state, and the cold equilibrium state to the $|\downarrow\rangle$ state. The resetting and initial state preparation of the spin can be finished in less than 5 μs, thus ensuring that the time spent in the heating and cooling strokes is negligibly small compared to the total time τ of one Otto cycle, which is usually around 100 μs. In addition, the T_2^* time is 10 ms, and the heating rate is 240 quanta/s, much longer than the maximal $N\tau$ of the experiment.

During the expansion and compression strokes of one Otto cycle, a laser beam with wavelength of 729 nm is used to drive the resonant transition between the two-level states $\{|\uparrow\rangle, |\downarrow\rangle\}$, and to drive the coupling of the qubit to phonons, where the relative qubit-phonon coupling strength is decided by $\eta\Omega$. In the experiment, we apply an acousto-optic modulator (AOM) connected to power-amplified signal sources to induce laser field to the ion, where the signal is sourced by an arbitrary waveform generator (AWG)[9]. By programming the AWG with desired waveforms, we generate the special three-color light field that can perform frequency scanning and intensity modulation at the same time, as shown in Fig. S1. When it is needed to introduce the STA techniques, we would add another carrier component to the three-color light field with a $-\pi/2$ phase difference to $H_E(0) = \frac{\Omega}{2}\sigma_x$, as described in Eq. (S12), whose intensity and frequency are also programmed by AWG.

To make the analysis of the STA cost with the improve-

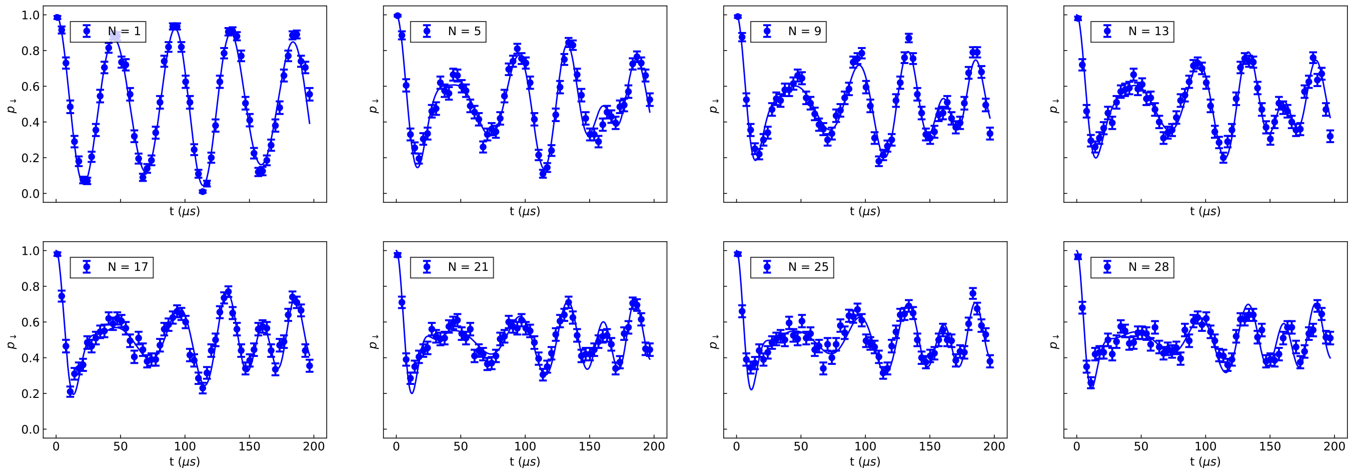

FIG. S4. Sample data for the measurement of $p_\downarrow$ state population at time t after different number of successive Otto cycles N, with $\Omega = 2\pi \times 0.159$ MHz, $\omega_z = 2\pi \times 2.0338$ MHz, $v_0 = \omega = 2\pi \times 0.075$ MHz and $\tau = 119$ μs. Blue circles are the measured average $p_\downarrow$ state population of the engine with STA scheme. Error bars correspond to 1 standard deviation of the normal distribution from the raw data which samples 200 times. Solid lines represent the corresponding numerical simulations.

ment it brings, we measured the amplitude of the CD laser during compression and expansion strokes by detecting its laser intensity onto a photo diode (PD). With a clock-synchronized oscilloscope, we are able to collect and analyze the intensity of the CD laser at different times in one Otto cycle. The intensity of the carrier laser at the same time is also measured through PD and oscilloscope. As shown in Fig. S3, the sampling interval of the oscilloscope is 1 μs, and the number of samples per point is 2000. By dividing the amplitude of the CD laser by the corresponding amplitude of the carrier laser, we calculated that the time-averaged integration value of this proportion only takes 2.6(2)%, while the corresponding numerical simulation result of this value is 2.5%.

The numerical simulations including experimental imperfections are performed by solving the Lindblad master equation to account for the influence of the environment

$$\dot{\rho}(t) = -\frac{i}{\hbar}[H(t), \rho(t)] + \sum_n \frac{1}{2}[2C_n\rho(t)C_n^\dagger - \rho(t)C_n^\dagger C_n - C_n^\dagger C_n \rho(t)], \quad \text{(S13)}$$

where the C_n are the Lindblad dissipators. In our simulations, the three dissipators respectively consist of the phonon decoherence term $\sqrt{\Gamma_1}a^\dagger a$ with the decoherence rate $\Gamma_1 = 100$ Hz, the phonon heating term $\sqrt{\Gamma_2}a$ and $\sqrt{\Gamma_2}a^\dagger$ with the heating rate $\Gamma_2 = 240$ Hz, and the spin decoherence term $\sqrt{\Gamma_3}\sigma_z$ with the decoherence rate $\Gamma_3 = 250$ Hz, measured in separate experiments.

B. Measurement of the average phonon number

The experiment begins with the 397 nm laser in conjunction with a repumper laser at 866 nm to Doppler cool the ion motion, then a series of controlled 729 nm laser and 854 nm repumper laser pulses are used to sideband cool the ion motion to the ground state $|0\rangle$ and initialize the spin state to $|\downarrow\rangle$[8]. We then apply a sequence of 729 nm laser pulses in conjunction with 854 nm repumper laser to drive several successive Otto cycles. We stop the engine evolution after a certain number of Otto cycles, and measure the average phonon number, which corresponds to the work produced by the heat engine on the external system. To measure the average phonon number, we initialize the two-level state to $|\downarrow\rangle$ state with negligible effect on the phonon state, then we drive the blue sideband transition between $|\downarrow, n\rangle$ and $|\uparrow, n+1\rangle$ for various time interval t. The interaction Hamiltonian of the blue sideband (bsb) transition is:

$$H_{int}^{bsb} = \frac{\Omega^{bsb}}{2}(e^{-i(\delta t+\phi_L)}\sigma_+ \exp\{i\eta(ae^{-i\omega_z t}+a^\dagger e^{i\omega_z t})\}+h.c.), \quad \text{(S14)}$$

where $\delta = \omega_L - \omega_0 = \omega_z$. In this interaction picture, the wavefunction of the tensor space consisting of spin and momentum can be written as $\Phi(t) = \sum_{m_z=\uparrow,\downarrow}\sum_{n=0}^{\infty} C_{m_z,n}(t)\,|m_z, n\rangle$, where $|m_z\rangle$ and $|n\rangle$ are the time-independent spin and motion eigenstates. If the transitions are coherently driven between $|\downarrow, n\rangle$ and $|\uparrow, n+1\rangle$, the coefficients $C_{m_z,n}(t)$ are given by Schrödinger's equation $i\partial\Phi/\partial t = H_{int}^{bsb}\Phi$ as[10]:

$$\dot{C}_{\uparrow,n+1} = e^{i\phi_L}\Omega_{n+1,n}^{bsb} C_{\downarrow,n}, \quad \text{(S15)}$$

$$\dot{C}_{\downarrow,n} = e^{-i\phi_L}\Omega_{n+1,n}^{bsb} C_{\uparrow,n+1}, \quad \text{(S16)}$$

where $\Omega_{n+1,n}^{bsb} = \Omega^{bsb}|\,\langle n+1|\, e^{i\eta(a+a^\dagger)}\,|n\rangle\,|$. For an initial state of $\Phi(0) = \sum_{n=0}^{\infty} C_{\downarrow,n}(0)\,|\downarrow, n\rangle$, the coefficients

$C_{m_z,n}(t)$ during evolution can be solved as:

$$\Phi(t) = \sum_{n=0}^{\infty} C_{\downarrow,n}(0)(\cos(\Omega^{bsb}_{n+1,n}t)\,|\downarrow,n\rangle + e^{i\phi_L}\sin(\Omega^{bsb}_{n+1,n}t)\,|\uparrow,n+1\rangle), \tag{S17}$$

with

$$p_\downarrow(t) = \sum_{n=0}^{\infty} p_n \cos^2(\Omega^{bsb}_{n+1,n}t). \tag{S18}$$

Thus by fitting the resultant $|\downarrow\rangle$ state population, we can reconstruct the population p_n of different phonon eigenstates $|n\rangle$. Here, Eq. (S18) is used as the fitting function to obtain the possible results of phonon population distribution $p(n)$ through curve fitting. When estimating the error bar of the fitting results, we make a common assumption of independent and identically distributed Gaussian noise of the experimental data, and the fitted parameters also follow a joint Gaussian distribution. In Fig. S4, we use the data collected in the multiple-cycle quantum engine experiment with STA scheme as an example to illustrate our measurement results of $p_\downarrow(t)$, which correspond to the $\bar{n}_{STA}(N)$ results as shown in Fig. 2 in the main text.

In the phonon number distribution fitting, in order to avoid the occurrence of overfitting, we use the lowest cut-off number n_{max} and ensure that the total occupation of all the phonon eigenstates is above 95%, that is $\sum_{n=0}^{n_{max}} p(n) \geq 0.95$[11]. Our principle of choosing the proper n_{max} is to make the fitting error bar of the average phonon number smaller, while the total occupation still exceeds 95%. Here, we take the phonon number distribution of the $\bar{n}_{STA}$ result at $N = 28$, which has the largest average phonon number in this experiment, as an example to show how we choose a proper n_{max}. As shown in Fig. S5(a), according to the numerical simulations, for this phonon state, when the cut-off number reaches $n_{max} = 8$, the total occupation $\sum_{n=0}^{n_{max}} p(n)$ starts to be above 95.5%. In the phonon number distribution fitting, the extracted average phonon number is 2.92, while the fitting error of the average phonon number is 0.24. When we continue to increase the cut-off number to $n_{max} = 9$, with the total occupation is around 96.5%, the results of the phonon number distribution are similar to the results of $n_{max} = 8$, and the average phonon number is 3.14(0.28), as shown in Fig. S5(b). However, if we want to further increase the total occupation to be above 99.5%, since for this phonon state, its high-phonon population only accounts for a small proportion of the total occupation, we need to increase the cut-off number to $n_{max} = 19$. As shown in Fig. S5(c), the error bar of the state occupation after $n = 8$ becomes dramatically large, which results in the curve fitting result of the average phonon number to be 5.6(8.3), indicating that overfitting occurs. Hence, we chose the cut-off number as $n_{max} = 8$. In order to ensure that the average phonon number extracting method is consistent for all the experimental data at different average phonon numbers, we uniformly set our principle of choosing the proper n_{max} to be that make sure the total occupation exceeds 95%.

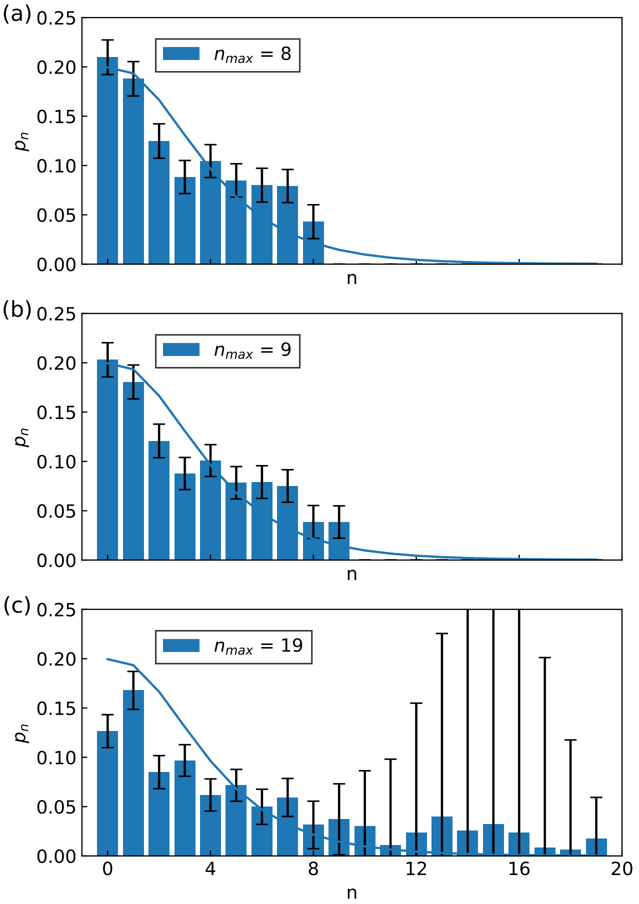

FIG. S5. Phonon number distribution p_n with different cutoff number n_{max}. (a) Phonon number distribution with cut-off number $n_{max} = 8$, the extracted average phonon number is 2.9(2) while the total occupation $\sum_{n=0}^{n_{max}} p(n)$ is above 95.5%. (b) Phonon number distribution with $n_{max} = 9$, the extracted average phonon number is 3.1(3) and $\sum_{n=0}^{n_{max}} p(n) > 96.5\%$. (c) Phonon number distribution with $n_{max} = 19$, the extracted average phonon number is 6(8) and $\sum_{n=0}^{n_{max}} p(n) > 99.5\%$. The bar charts are the results of curve fitting. Error bars correspond to 1 standard fitting error of the phonon state population. Solid lines represent the corresponding numerical simulations.

On the basis of choosing a proper cut-off number n_{max}, another method that can be added to further reduce the fitting error is to give the function form of the phonon number distribution $p_n = f(n)$, thereby reducing the number of fitting parameters and avoiding the occurrence of overfitting. For example, an ions motional state under background heating is given by a thermal distribution $p_n = (\frac{\bar{n}}{\bar{n}+1})^n$ with the average phonon number of $\bar{n}$. In this experiment, it can be proved that the distribution of work driven by the Hamiltonian $H_B + H_{EB}(t)$ [Eq. (S1)]

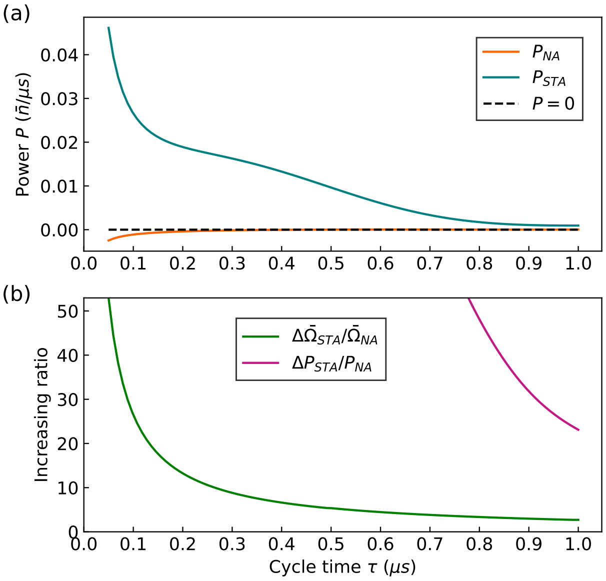

FIG. S6. Simulated engine power P and increasing ratio of STA techniques at different cycle time τ, with $\Omega = 2\pi \times 0.159$ MHz, $\omega_z = 2\pi \times 2.0423$ MHz, $v_0 = \omega = 2\pi \times 0.075$ MHz and $N = 1$. (a) Orange(Teal) solid line $P_{NA}(P_{STA})$ represents the numerical simulation results of engine power of the non-adiabatic(STA) engine. The black dashed line represents the case where the output power of the heat engine is zero. (b) Pink(Green) solid line $\Delta\bar{P}_{STA}/\bar{P}_{NA}(\Delta\bar{\Omega}_{STA}/\bar{\Omega}_{NA})$ represents the increasing ratio of engine power(laser amplitude).

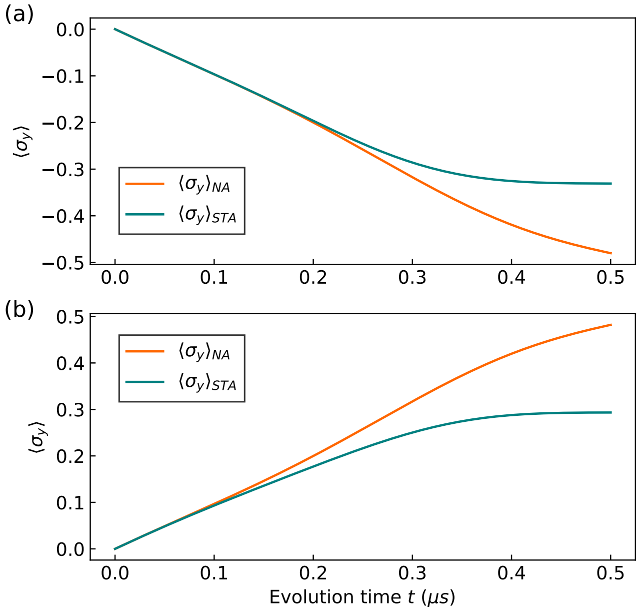

FIG. S7. Simulation results of the suppression effect of STA techniques on the expected population along the σ_y direction at different evolution time t, with cycle time $\tau = 1$ μs, $\Omega = 2\pi \times 0.159$ MHz, $\omega_z = 2\pi \times 2.0423$ MHz, $v_0 = \omega = 2\pi \times 0.075$ MHz and $N = 1$. (a) Orange(Teal) solid line $\langle\sigma_y\rangle_{NA}(\langle\sigma_y\rangle_{STA})$ represents the numerical simulation results of the expected population along the σ_y direction of the non-adiabatic(STA) engine during isentropic expansion. (b) Orange(Teal) solid line $\langle\sigma_y\rangle_{NA}(\langle\sigma_y\rangle_{STA})$ represents the numerical simulation results of $\langle\sigma_y\rangle$ for non-adiabatic(STA) engine during isentropic compression.

can be decomposed into a series of superpositions of exponential functions, and the relative coefficients of different series are determined by the initial state[3]. Therefore, for the experimental results that can already be proved in the simulations that its high-phonon population distribution has the form of an exponential function, we uniformly assume that it has the function form of $p_{n>n_0} = A\exp([B(n-n_0)] + C$, where $0 < n_0 < n_{max}$.

The reason why the total occupation only exceeds 95% may be due to the state preparation and measurement error. In the experiment, the motional heating rate in our setup is around 240 phonon/s, which results in the motional decoherence effect. Other sources of errors can be from the fluctuation of the trap frequency ω_z, spin population detection error, and from the phonon number fitting since some noise in the signals may be incorrectly recognized as a high-phonon population and cause the fitting error.

III. EFFECTS OF THE SHORTCUT

A. Suppression of the irreversible entropy production

Since non-adiabatic transitions are well-known sources of entropy production that reduce the efficiency of thermal machines[12], the dynamics of the quantum engine can be sped up with the help of STA techniques to suppress the unwanted non-adiabatic transitions, thereby reducing the associated production of entropy[13,15]. The suppression of this irreversible entropy production with the help of STA techniques can be observed when the working medium experiences a fast driving during the work strokes[7]. Figure S6 displays the numerical simulation results of engine power during a fast driving, where the parameters are set the same as Fig. 4 in the main text. We observe in Fig. S6(b) that the output power of the STA engine has a finite value above $P = 0$ at very short driving times $\tau < 0.1$ μs, while the non-adiabatic engine is unable to generate work output[7].

The reason why we didn't collect and show this kind of experimental results in the main text is that, as mentioned before in the experimental setup, we use the programmed laser pulses to drive the Otto cycles, so due to the limitation of sampling rate and the existence of rising edge from AOMs, it is impossible for us to accomplish such a fast driving in experiments. Besides, the resetting and initial state preparation of the spin takes about $2 \sim 5$ μs, which means that for such a fast-driving engine, the heating and cooling strokes will take up most

of the time of one cycle, which is also contrary to the common definitions of the Otto cycle. For the above reasons, in our experiments, the total time of one Otto cycle is usually selected to be around 100 μs, in which the changing speed is slow so that the evolution of the working medium becomes quasistatic[6]. Therefore, we can only show the numerical simulation results of this irreversible entropy production phenomenon, but cannot directly demonstrate it in experiments.

B. Suppression of the nonadiabatic transitions

In the experiments, we add a counterdiabatic Hamiltonian H_{CD} to the engine Hamiltonian H_E in order to suppress the nonadiabatic transitions and reduce coherent oscillations along the σ_y direction. The suppression of detrimental nonadiabatic transitions with STA techniques can be observed by calculating the expected population along the σ_y direction, which is denoted as $\langle\sigma_y\rangle$, during the isentropic expansion and isentropic compression of one Otto cycle. Figure S7 displays the numerical simulation results of $\langle\sigma_y\rangle$ during the isentropic expansion and isentropic compression of a fast driving with cycle time $\tau = 1$ μs, where the parameters are set the same as Fig. S6. We observe in Fig. S7 that at the start of the engine's expansion and compression process, the initial population along the σ_y direction is always 0 since we reset the two-level state to the spin-up $|\uparrow\rangle$ (spin-down $|\downarrow\rangle$) state during the heating and cooling process. But with the increasing of the evolution time t, the expected population of $\langle\sigma_y\rangle$ will gradually become non-zero, resulting in coherent oscillations along the σ_y direction. After adding the STA scheme, the absolute value of the expected population along the σ_y direction of the STA engine $\langle\sigma_y\rangle_{STA}$ is significantly reduced compared with the non-adiabatic engine $\langle\sigma_y\rangle_{NA}$, and the gap between them is also widening with the increase of evolution time t. Therefore, at the end of the engine's expansion and compression process, the expected population along the σ_y direction of the STA engine is only around 0.3, while that of the non-adiabatic engine reaches about 0.5, which proves that the STA scheme can effectively suppress the nonadiabatic transitions and reduce coherent oscillations along the σ_y direction.

In the cycles, we perform a sequential coherent operation U_j interleaved by two-level projection operation $P_i = |i\rangle\langle i|$, with $j = \{e, c\}$ represents either expansion or compression and $i = \{\uparrow, \downarrow\}$. The corresponding evolution operator after N cycle is:

$$U_N = (U_e P_\uparrow U_c P_\downarrow)^N. \tag{S19}$$

Since the projection operator is invariant under the rotation along the z axis, i.e. $P_i = e^{i\phi\sigma_z} P_i e^{-i\phi\sigma_z}$ for any ϕ, one can arbitrarily apply such transformation in between the operators U_j without affecting the results. Thus, the two-level coherence is heavily interrupted by each projection. In fact, compared with the case that a desired spin thermal state is prepared between the coherent process, in a single-shot manner the state is indeed coherent however without knowledge with respect to the external drive, which resembles the condition of the above analysis. The motional coherence comes from, instead, the relative phase within $\sigma_y(a + a^\dagger)$, which is intact from the above process, resulting in the build-up of the motional coherence. Thus, we predict the coherence for the qubit component at the beginning of each cooling(heating) step does not play a role in the process.

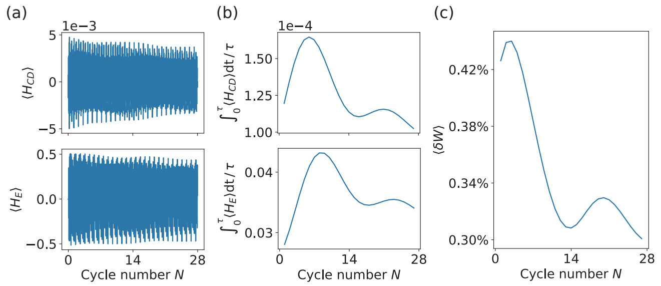

FIG. S8. Simulation of an alternative cost metric of the shortcut protocol. (a) Expectation value of the counterdiabatic driving Hamiltonian H_{CD} and of the quantum engine Hamiltonian H_E as a function of the cycle number N: $\langle H_{CD}\rangle$ is about a factor hundred smaller than $\langle H_E\rangle$ for the parameters of the experiment. (b) Time averages of both expectation values at the end of each cycle. (c) Ratio of the two averages at the end of each cycle. Parameters are $\Omega = 2\pi \times 0.159$ MHz, $\omega_z = 2\pi \times 2.0338$ MHz and $v_0 = \omega = 2\pi \times 0.075$ MHz.

C. An alternative cost of the shortcut

Many metrics have been proposed to quantify the cost of shortcut protocols[14]. We here evaluate the cost of the counterdiabatic driving by numerically calculating the ratio of the time average of the expectation of the shortcut driving driving Hamiltonian H_{CD} to that of the quantum engine Hamiltonian H_E following Ref.[15]

$$\langle\delta W\rangle = \int_0^\tau \langle H_{CD}\rangle(t)dt \Big/ \int_0^\tau \langle H_E\rangle(t)dt. \tag{S20}$$

The result shown in Fig. S8 indicates that the ratio is of the order of 0.4%, which is again a small number. As before, we refrain from tomographically reconstructing the qubit density matrix during the cycle in order to preserve its quantum properties.

IV. WORK OSCILLATIONS AND QUANTUM COHERENCE

In this section, we relate the oscillations of the work output seen in Fig. 2 to the presence of quantum coherence in the energy basis of the harmonic oscillator.

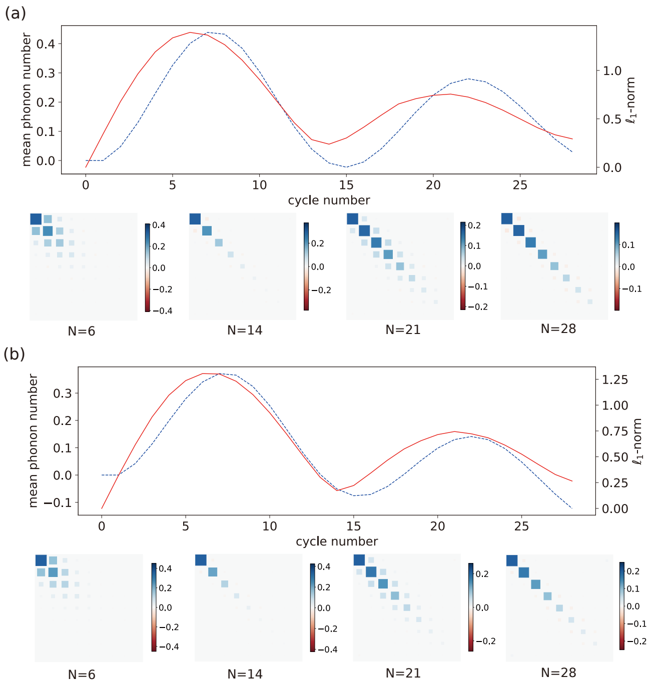

FIG. S9. Simulated phonon density matrix and its ℓ_1-norm of coherence (red solid), (a) without and (b) with counter-diabatic driving. The density matrices at the end of 6, 14, 21, 28 cycles are shown; the color and the size of the blocks represent the absolute value of the matrix elements. We also show the numerically simulated mean phonon number, minus the non-oscillating classical contribution, (blue dashed) whose oscillations are directly related to those of ℓ_1-norm of coherence. Parameters are $\tau = 120\ \mu$s, $\Omega = 2\pi \times 0.159$ MHz, $\omega_z = 2\pi \times 2.0338$ MHz and $v_0 = \omega = 2\pi \times 0.075$ MHz.

To that end, we numerically simulate the density matrix of the phonon states for the parameters of the experiment, and evaluate the l_1-norm of coherence[16], that is, the summation of the absolute value of non-diagonal elements $||\rho||_{\ell_1} = \sum_{i\neq j} |\rho_{i,j}|$. Note that the density matrix can, in principle, be tomographically reconstructed experimentally[1]. However, the large number of levels involved in our experiment, as well as the small value of the off-diagonal matrix elements compared to the error bars of the mean phonon numbers, make such full reconstruction challenging in practice.

Figure S9 shows the evolution of the phonon density matrix as a function of the cycle number, without (a) and with (b) shortcut. We observe in both cases that the diagonal elements (populations) spread to higher phonon states as the harmonic battery is charged, and that the nondiagonal elements (coherence) periodically increase and decrease, quantifying intercycle quantum coherence. In addition, Fig. S9 reveals that the oscillations of the work output (blue dashed) are directly connect to those of the l_1-norm of coherence (red solid). The oscillation of the mean phonon number can thus be attributed to intercycle quantum coherence in the energy basis.

* These authors contribute equally.
† Corresponding author; Email: krehan2010@yahoo.com
‡ Corresponding author; Email: eric.lutz@itp1.uni-stuttgart.de
§ Corresponding author; Email: yiheng@ustc.edu.cn
¶ Corresponding author; Email: djf@ustc.edu.cn

1. D. Leibfried, R. Blatt, C. Monroe, and D. Wineland, Quantum dynamics of single trapped ions, Rev. Mod. Phys. **75**, 281 (2003).
2. G. Watanabe, B. P. Venkatesh, P. Talkner, and A. del Campo, Quantum Performance of Thermal Machines over Many Cycles, Phys. Rev. Lett. **118**, 050601 (2017).
3. P. Talkner, P. S. Burada, and P. Hänggi, Statistics of work performed on a forced quantum oscillator, Phys. Rev. E **78**, 011115 (2008).
4. S. Chand and A. Biswas, Single-ion quantum Otto engine with always-on bath interaction, EPL **118**, 60003 (2017).
5. M. V. Berry, Transitionless quantum driving, J. Phys. A **42**, 365303 (2009).
6. O. V. Ivakhnenko, S. N. Shevchenko, and F. Nori, Nonadiabatic Landau-Zener-Stückelberg-Majorana transitions, dynamics, and interference, Phys. Rep. **995**, 1 (2023).
7. B. Cakmak and O. E. Mustecaplioglu, Spin quantum heat engines with shortcuts to adiabaticity, Phys. Rev. E **99**, 032108 (2019).
8. Ch. Roos, Th. Zeiger, H. Rohde, H. C. Nägerl, J. Eschner, D. Leibfried, F. Schmidt-Kaler, and R. Blatt, Quantum state engineering on an optical transition and decoherence in a Paul trap, Phys. Rev. Lett. **83**, 4713 (1999).
9. M. Zhang, X. Yuan, Y. Li, X. Luo, C. Liu, M. Zhu, X. Qin, C. Zhang, Y. Lin, and J. Du, Observation of Spin-Tensor Induced Topological Phase Transitions of Triply Degenerate Points with a Trapped Ion, Phys. Rev. Lett. **129**, 250501 (2022).
10. D. J. Wineland, C. Monroe, W. M. Itano, D. Leibfried, B. E. King, and D. M. Meekhof, Experimental Issues in Coherent Quantum-State Manipulation of Trapped Atomic Ions, J. Res. Natl. Inst. Stand. Technol. **103**, 259 (1998).
11. M.-L. Cai, Z.-D. Liu, W.-D. Zhao, Y.-K. Wu, Q.-X. Mei, Y. Jiang, L. He, X. Zhang, Z.-C. Zhou and L.-M. Duan, Observation of a quantum phase transition in the quantum Rabi model with a single trapped ion, Nat. Commun. **12**, 1126 (2021).
12. O. Abah and E. Lutz, Energy efficient quantum machines, EPL **118**, 40005 (2017).
13. O. Abah and E. Lutz, Performance of shortcut-to-

adiabaticity quantum engines, Phys. Rev. E **98**, 032121 (2018).

[14] D. Guery-Odelin, A. Ruschhaupt, A. Kiely, E. Torrontegui, S. Martinez-Garaot, and J. G. Muga, Shortcuts to adiabaticity: Concepts, methods, and applications, Rev. Mod. Phys. **91**, 045001 (2019).

[15] O. Abah and M. Paternostro, Shortcut-to-adiabaticity Otto engine: A twist to finite-time thermodynamics, Phys. Rev. E **99**, 022110 (2019).

[16] T. Baumgratz, M. Cramer, and M. B. Plenio, Quantifying Coherence, Phys. Rev. Lett. **113**, 140401 (2014).
